# Moral injury in a VUCA healthcare environment: ethical complexities and professional challenges for medical teams

**DOI:** 10.1186/s12913-026-14664-2

**Published:** 2026-05-08

**Authors:** Lior Naamati-Schneider, Hagar Binoun-Chaki, Paula Feder-Bubis, Shir Daphna-Tekoah

**Affiliations:** 1 Health Systems Management Department, Jerusalem Multidisciplinary College, Jerusalem, Israel; 2 Faculty of Social Work, Ashkelon Academic College, Ashkelon, Israel; 3 Department of Health Policy and Management, Ben-Gurion University of the Negev, Beer Sheva, Israel

**Keywords:** Moral injury, Healthcare professionals, VUCA framework, Ethical dilemmas, Professional identity, Emotional distress, Resilience, Patient-centered care, Digital transformation in healthcare

## Abstract

**Background:**

Healthcare systems operate within a VUCA (Volatile, Uncertain, Complex, and Ambiguous) environment, shaped by economic, demographic, and systemic transformations. These rapid and unpredictable changes create ethical challenges, resource constraints, and heightened emotional and moral distress for healthcare professionals. The increasing complexity of care delivery, shifting institutional priorities, and external pressures contribute to moral injury, impacting professionals’ ability to provide patient-centered care while maintaining their ethical and professional integrity.

**Methods:**

This qualitative study aimed to explore how healthcare professionals experience and cope with moral injury in a VUCA healthcare ecosystem. Through 35 semi-structured interviews, the study explores how healthcare professionals experience and cope with moral injury in such a dynamic healthcare ecosystem. The research uses an abductive analysis guided by the VUCA framework to examine the systemic roots of moral conflict.

**Results:**

The analysis identified six themes highlighting how instability, unpredictability, ambiguity, and systemic overload shape clinical decision-making, emotional burden, and ethical distress. Participants described moral injury as emerging from the misalignment between professional values and institutional demands, intensified by resource shortages, role ambiguity, and crisis normalization. These pressures affect professionals’ well-being, compromise ethical integrity, and contribute to long-term psychological consequences.

**Conclusion:**

The findings emphasize the need to move beyond individual-level resilience strategies and focus on systemic reforms. Strengthening institutional support structures—including ethical leadership, reflective spaces, and alignment between organizational policy and professional ethics—is essential for protecting both clinicians’ integrity and care quality in today’s complex healthcare landscape.

**Supplementary Information:**

The online version contains supplementary material available at 10.1186/s12913-026-14664-2.

## Introduction

In recent decades, rapid and unpredictable changes have shaped a world characterized by volatility, uncertainty, complexity, and ambiguity (VUCA) [1]. This evolving landscape poses significant challenges to various sectors, including healthcare. Healthcare systems worldwide must continually adapt to demographic shifts, economic pressures, technological advancements, and socio-political transformations, all of which influence the broader ecosystem in which they operate [2–5]. These forces not only reshape healthcare systems but also drive the rising demand for medical services, exacerbating financial and resource constraints, increasing operational pressures, and generating ethical dilemmas that impact medical teams at multiple levels [6–7].

Broader structural challenges also contribute to moral distress among healthcare professionals. Economic constraints and rising healthcare expenditures threaten the long-term sustainability of medical services, leading to increasing workloads, workforce shortages, and shifts in organizational priorities [8–9]. Healthcare organizations must continuously adapt their managerial, financial, and operational strategies to remain viable in this turbulent environment [3, 8, 10]. However, these adaptations often create conflicts between efficiency-driven policies and professional values, placing frontline clinical teams in ethically complex situations that may contribute to emotional exhaustion, moral distress, and even moral injury.

Among the most significant transformations is the accelerated digitalization of healthcare, particularly following the COVID-19 pandemic. Technologies such as telemedicine, mobile health platforms, virtual reality (VR), and augmented reality (AR) have improved access and reduced the physical burden on facilities [11–13]. However, these health services modalities also raise concerns related to patient engagement, equity, decision-making complexity, and team cohesion [14–15]. Integrating these modalities requires attention to ethical implications, data security, and systemic readiness, as technological change alone cannot resolve the emotional and ethical burdens professionals face [16–17].

Together, these structural, technological, and economic pressures, when examined through the lens of the VUCA framework, highlight how moral and ethical conflicts are intensified in contemporary healthcare. Healthcare professionals must navigate these intersecting changes while managing ethical dilemmas that test both institutional resilience and personal well-being. This study explores how healthcare professionals experience and respond to these challenges, offering insight into the impact of system-wide transformations on moral injury. By mapping personal experiences and identifying key dilemmas, the research aims to inform policy and practice strategies that support healthcare professionals and promote resilience in an unpredictable and demanding healthcare landscape.

## Theoretical background

### The VUCA framework

Originally conceptualized by the U.S. Army War College to describe the unpredictable global landscape after the Cold War [18], the term VUCA was formally introduced in the late 1990s and gained broader recognition following the 9/11 terrorist attacks in 2001 [19]. Over time, the concept has expanded beyond its military context and is now widely applied across various sectors. Business leaders, scholars, and policymakers use VUCA to describe the complex and rapidly evolving global environment, shaping decision-making and strategic approaches in an era of disruption [20–23]. More broadly, VUCA has become a crucial framework for understanding complex challenges that shape decision-making and policy development in multiple domains [21, 23], becoming the “new normal” [24].

*Volatility* refers to rapid, unpredictable fluctuations over an unspecified duration [25]. It is related to continuous instability, such as price fluctuations in stock markets or shifts in supply and demand following economic shocks. Additionally, workforce trends, such as Millennials frequently changing jobs to pursue new opportunities, contribute to volatility in labor markets [26].

*Uncertainty* arises when cause and effect are known, but the ability to assess the significance of a situation is lacking [25]. As volatility increases, continuously shifting data makes it harder to anticipate outcomes [24]. Uncertainty is prevalent in global business environments, where exposure to unpredictable elements, such as political instability, regulatory changes, economic fluctuations, social unrest, natural disasters, competition, and disruptive innovations, creates heightened risks [27].

*Complexity* refers to situations involving numerous interdependent variables, making them difficult to analyze and manage [25]. Rapid technological advancements have accelerated information flow, creating intricate, multilayered data networks with unforeseen consequences [24]. Globalization has further amplified complexity, as organizations must navigate regulatory frameworks, cultural differences, and diverse educational systems across multiple countries. Multinational corporations, in particular, struggle to maintain legitimacy while addressing competing legal, social, and institutional demands [28].

*Ambiguity* represents the highest level of uncertainty, an “unknown-unknown” where neither causes nor outcomes are clear [25]. In ambiguous situations, no historical precedent exists to provide guidance, making predictions and decision-making highly uncertain. As volatility, uncertainty, and complexity intensify, ambiguity becomes inevitable, further complicating strategic planning [24].

Contemporary systems exhibit heightened VUCA conditions through global crises, economic instability, cyber risks, climate change, and political upheaval. Social media accelerates both information and misinformation, influencing public perceptions and contributing to instability [29]. Events such as Brexit, trade conflicts, and the COVID-19 pandemic illustrate how VUCA dynamics disrupt multiple sectors, including healthcare, supply chains, and labor markets [30–36]. Remote work adoption and organizational adaptation pressures further highlight how VUCA conditions shape managerial and operational realities [37].

Global institutions increasingly acknowledge these disruptions while struggling to respond effectively [38]. Yet VUCA conditions also create opportunities for innovation and resilience building as systems adapt to digital transformation, geopolitical change, and shifting social structures [31, 39–40].

### The VUCA healthcare ecosystem

Healthcare systems face VUCA pressures that intensify existing structural vulnerabilities. Rising life expectancy, chronic disease prevalence, and public expectations drive increased demand, while financial resources fail to keep pace [6, 9, 41]. As a result, healthcare organizations navigate a turbulent environment shaped by regulation, workforce shortages, technological changes, and competing market forces [3, 10].

The COVID-19 pandemic amplified these dynamics. Healthcare organizations confronted unfamiliar clinical challenges, overwhelming patient loads, suspended elective care, and workforce depletion [42]. These disruptions created financial instability and reputational risks as clinics faced reduced activity, extended wait times, and staffing constraints [43].

The crisis accelerated digital transformation, compelling organizations to adopt telemedicine, virtual care, and advanced digital systems to navigate uncertainty [44–45]. Yet despite their benefits, these technologies introduce ethical and operational challenges related to data security, prioritization of care, and patient confidentiality [46–47]. The shift toward patient centered care and shared decision making further increases complexity by expanding providers’ responsibilities and ethical considerations [48–50].

Increasing demands for transparency and accountability require providers to continuously balance institutional obligations with professional ethics [46–47]. Many health systems have therefore revised managerial and financial strategies to address these evolving pressures [3, 10, 17].

### Ethical conflict, ethical judgment, and moral dilemmas as foundations of moral distress and moral injury in a VUCA healthcare environment

VUCA conditions intensify ethical conflict within healthcare systems by placing professionals in situations where competing values and institutional constraints collide [3, 5, 46–47]. Healthcare ethics relies on beneficence, autonomy, non maleficence, and justice [51]. Moral dilemmas arise when it is impossible to satisfy all ethical obligations simultaneously, particularly in settings defined by resource shortages, time constraints, or conflicting policies [52, 53].

Ethical judgment becomes more complex under systemic inefficiencies, economic pressures, and regulatory uncertainties. When institutional requirements conflict with professional values, providers may experience moral distress and internal value conflict [54–55]. Viewed through the VUCA lens, instability and rapidly changing expectations amplify psychological burden and reduce professionals’ sense of agency [46–47].

### Moral injury as an outcome of ethical conflict and distress

Moral injury describes the psychological harm that occurs when individuals participate in, witness, or are unable to prevent actions that violate their deeply held moral beliefs [56–57]. In contrast to post traumatic stress disorder (PTSD), which is rooted in fear based trauma, moral injury arises from guilt, shame, and moral disorientation. These experiences are highly relevant in healthcare, where professionals routinely make ethically challenging decisions while working within systemic constraints such as limited resources, bureaucratic demands, or organizational inefficiencies [58–60]. Exposure to morally injurious events has been associated with increased risk of PTSD symptoms, illustrating the psychological toll of unresolved ethical distress [61].

Although the concept of moral injury in healthcare has gained attention only in recent years, it is often confused with burnout. Burnout traditionally reflects emotional exhaustion, diminished efficacy, and overload caused by sustained work pressures [62]. Extensive research has documented high levels of burnout, stress, and trauma among healthcare workers, largely attributed to intense workloads and organizational demands [63–64]. However, scholars increasingly argue that burnout alone does not fully capture the ethical and emotional burden experienced by clinicians. Talbot and Dean [65] suggest that moral injury offers a more accurate framework for understanding situations in which professionals feel compelled to act in ways that conflict with their moral values.

Further support comes from research on medical trainees. Meacham [66] demonstrates how Potentially Morally Injurious Events (PMIEs) challenge caregivers’ integrity, sense of purpose, and professional identity, illustrating the distinct mechanisms through which moral injury develops. These insights raise questions about the suitability of traditional burnout focused interventions, such as resilience training. Such approaches may inadvertently intensify distress by personalizing a problem that stems from systemic and ethical constraints rather than from individual coping deficits [66]. Consequently, scholars call for support strategies that address structural and ethical dimensions of healthcare work, particularly in high stakes environments [65](Talbot & Dean, 2018).

Moral injury is especially pronounced in life and death decision making. Healthcare professionals frequently operate under resource scarcity, operational inefficiencies, and institutional limitations that heighten emotional strain and reduce moral clarity [67–69]. The COVID 19 pandemic exemplified this dynamic. Clinicians confronted overwhelming caseloads, triage dilemmas, workforce shortages, and personal health risks, all of which intensified moral distress and contributed to long term psychological consequences [70–73].

Broader systemic characteristics add further layers of complexity. Structural inefficiencies, chronic understaffing, and conflicting institutional priorities diminish professional autonomy and erode clinicians’ capacity to act according to their ethical commitments. Studies consistently show that such pressures compromise moral integrity, reinforcing ethical conflict as a central mechanism in the development of moral injury [74]. Unlike secondary trauma or vicarious traumatization, which emerge from exposure to others’ suffering, moral injury stems specifically from ethical violations or the inability to uphold core moral values [75–78].

Despite increasing interest in clinician well being, the phenomenon of moral injury remains insufficiently examined in healthcare settings characterized by instability and high risk. Its origins lie in structural and ethical challenges embedded within healthcare delivery rather than in personal resilience deficits. Addressing moral injury therefore requires organizational strategies that strengthen ethical practice, support moral agency, and provide safe spaces for ethical reflection. Such efforts are essential for preserving the integrity, motivation, and long term retention of healthcare professionals [65].

### Israel’s unique VUCA healthcare challenges and moral injury

While global VUCA forces shape healthcare systems worldwide, Israel faces an added layer of chronic instability due to ongoing political, social, and security challenges. The combination of global uncertainty with regional conflicts, terror threats, and emergency responses makes it a key case study for moral injury among healthcare professionals. Israeli medical teams operate in persistent uncertainty, frequently making high-stakes ethical decisions in mass trauma events, politically sensitive situations, and cases involving both victims and perpetrators. The compounded stress of responding to both local and global crises intensifies their emotional and ethical burdens.

Although based on Israel, this study offers broader insights into conflict-prone and high-risk environments worldwide. By examining systemic, societal, and professional contributors to moral distress, it provides a valuable perspective on how instability, unpredictability, and conflict impact medical ethics and workforce resilience across volatile global settings [2, 20, 74]. Findings will enhance understanding of how VUCA-driven pressures contribute to moral injury, offering valuable insights for future research and policy development to better support healthcare professionals facing ethical and systemic instability.

## Methods

### This study

This qualitative study explores moral injury among healthcare professionals, focusing on the systemic challenges, ethical dilemmas, and emotional burdens they face. Using an abductive approach, the study maps the broader healthcare ecosystem as a framework while uncovering emerging moral conflicts and injuries. It examines how systemic pressures, operational challenges, and internal moral dilemmas intersect through in-depth narratives and thematic analysis.

This study is part of a larger research project (2021–2025) examining the healthcare system environment in a VUCA-driven landscape. Participants included frontline workers: nurses, physicians, social workers, administrators, and hospital decision-makers, ensuring diverse perspectives on moral injury and operational complexities.

### Participants and sampling

This study included 35 physicians and nurses from large hospitals, selected for their scale, patient volume, and dynamic healthcare environment. The sample included 14 physicians, 15 nurses, and 6 participants in managerial or administrative roles. Representing various departments, genders, and cultural backgrounds, they provided a broad spectrum of experiences. Recruitment followed a snowball sampling approach, beginning with participants from different hospitals. Invitations were sent via email, and participants referred colleagues who met the study’s criteria, expanding the participant pool. The final sample size was determined by data saturation, when no new themes emerged in later interviews [79].

### Data collection and ethical considerations

Ethical approval was obtained from the ethics committee for Research on Human Subjects (IRB) at the Jerusalem Multidisciplinary Academic College before data collection (approval no. 691). All participants provided informed and voluntary consent prior to participation. All procedures involving human participants were conducted in accordance with relevant ethical guidelines and regulations, including the Declaration of Helsinki. Participants who provided informed consent took part in online interviews conducted via Zoom, which ensured accessibility despite demanding schedules. All meetings were password protected, participation was voluntary, and confidentiality and anonymity were strictly maintained.

Before each interview, participants reviewed a consent form outlining the study’s approval, their right to withdraw, and confidentiality measures. Interviews lasted approximately 45 min and were audio-recorded and transcribed verbatim following standard protocols [80]. Interviews were conducted by the authors or trained research assistants. To ensure consistency, team members received comprehensive training, including pre- and post-interview briefings to help them process both the data and their own emotional responses.

A semi-structured interview guide with open-ended questions was developed specifically for the purposes of this study to enable participants to share both personal and professional experiences in depth and to explore healthcare professionals’ experiences, ethical conflicts, and perceptions of moral injury within the healthcare system. The interview guide is available in the supplementary materials.

This format ensured consistency across interviews while preserving the richness and spontaneity of participants’ narratives. The interviews explored topics such as systemic challenges, ethical dilemmas, resource limitations, organizational constraints, and the ways in which evolving policies and organizational changes shaped clinical responsibilities and decision-making. This approach facilitated a nuanced and authentic exploration of moral injury within a complex and dynamic healthcare environment.

### Data analysis

This study employs abductive analysis [81] to examine moral injury in a VUCA-driven healthcare environment, integrating predefined theoretical themes with emerging insights regarding the interplay between systemic pressures and individual moral conflicts. The analysis began with an iterative review of interview transcripts, allowing researchers to immerse themselves in the data and identify key themes in VUCA challenges, ethical dilemmas, and moral distress. This method allowed for the discovery of previously unrecognized aspects of moral injury and the ways in which professionals adapt to VUCA-driven pressures. To ensure study trustworthiness, all research team members participated in coding the transcripts. The initial independent coding was followed by group discussions to compare findings, reconcile differences, and refine themes, ensuring consistency and accuracy. Once an agreed set of themes and sub-themes was established, the research team structured the findings within the VUCA framework, mapping moral injury onto volatility, uncertainty, complexity, and ambiguity within healthcare. The final step involved a comprehensive confirmation and refinement process, where themes were cross-checked throughout the dataset and existing theoretical models to ensure coherence and depth.

This structured abductive approach confirms existing theoretical constructs while uncovering new insights into how healthcare professionals navigate moral injury in a rapidly changing environment.

### Findings

The analysis revealed six main themes: *Volatility - Rapid Change and Instability*,* Complexity - Overload and Systemic Interdependence*,* Uncertainty –Unpredictability in Daily Operations*,* Ambiguity - Role Confusion and Ethical Uncertainty*,* Conflicts Between Professional Values and System Demands and Normalization of Crisis as a Coping Strategy.* Although these themes are analytically distinct, they are interconnected and reflect different dimensions of the VUCA environment. The structure of the findings follows the VUCA framework in order to differentiate between types of challenges, even when they are experienced simultaneously in practice. These findings underscore that the VUCA nature of the healthcare ecosystem deeply shapes clinicians’ daily work. The VUCA conditions manifest in their professional realities, generating ongoing tension between the need to adapt to systemic changes and the desire to uphold ethical and professional standards.

#### 1. Volatility - rapid change and instability

Many participants described their work environment as marked by frequent and rapid changes driven by changes in ecosystem needs, technological advancements, fluctuating policies, increasing patient demands, administrative demands, and shifts in healthcare protocols. These changes often occur without adequate preparation time, leading to feelings of instability and pressure. As Dikla, a nurse, explained: *The immense pressure and the need to respond quickly create a sense of urgency that accompanies every action*,* and it often feels like a race against the clock. There are changes all the time.*

This volatility shaped professionals’ perception of their environment as unpredictable, fostering a persistent sense of emergency. In this context, volatility refers to the speed and frequency of change, rather than to the uncertainty about future outcomes, which is discussed in the following theme. This state of flux resulted in what participants described as a “chronic crisis,” where professionals operated under the continuous expectation that a situation could escalate at any moment. This was not limited to acute emergencies; rather, it became embedded in their everyday reality, as Hadar, a nurse, reflected: *There are always emergencies here. Right now*,* we’re at war; before that*,* it was COVID*,* and in between*,* there’s never a break. Sometimes it’s terror attacks*,* sometimes injuries*,* diseases*,* we have everything*,* all the time.*

This ongoing volatility created a reality in which the line between routine operations and emergency response was increasingly blurred, further straining professionals’ capacity to regain control or establish a sense of stability in their daily work.

#### 2. Complexity - overload and systemic interdependence

Healthcare professionals described their work as highly complex, requiring constant integration of interrelated factors. They viewed the system as dynamic, where changes in one area quickly influence others, creating ripple effects that complicate daily decision making.

Balancing technological systems, administrative demands, and patient care often resulted in operational inefficiencies and information overload, ultimately reducing the time available for direct patient interaction. Professionals expressed frustration navigating this multifaceted environment, particularly when organizational procedures clashed with the practical realities of providing care, as Dina, a nurse, explained: *We need to remember that beyond all this technology*,* there is a patient. Sometimes*,* with all this progress*,* we forget that there is a patient. We have less face to face time with the patient*,* and you can feel it. And I am truly sorry about that.*

This systemic complexity, while intended to improve efficiency, often led to a sense of disconnection from the core mission of healthcare. As Alia, a nurse, observed: *Instead of being with the patients*,* I have to report to the computer every second.*

Ali, a doctor, described the difficulty of navigating the bureaucratic maze, even when trying to advocate for patients: In one of my roles in quality work, I try to bring order to the chaos, doctors, medications, special approvals, and *lab work required by HMOs. You can really get lost in it. And when I hit a wall with the system*,* trying to promote something that’s not within my authority*,* I feel like I’m failing to ease my patient’s burden and help them move forward.*

These reflections underscore how unmanaged complexity can obscure the human element at the heart of clinical work, further compounding professionals’ emotional and ethical burden. The need for constant adaptation left many feeling overburdened and constrained, which often conflicted with their professional judgment and commitment to patient care.

#### 3. Uncertainty – unpredictability in daily operations

Many participants described how the volatility and complexity embedded in the healthcare environment created not only rapid fluctuations but also a deeper layer of uncertainty that shaped professionals’ daily experience. Uncertainty refers to the lack of clarity regarding situations, expectations, and future outcomes, even when change itself is not necessarily rapid. Unlike volatility, which relates to the pace and instability of change, uncertainty refers to the difficulty of understanding what is happening in the present and anticipating what will come next. This theme emerged repeatedly in interviews, illustrating how unpredictability is woven into clinical practice.

Several professionals described difficulties not only in forecasting future developments but also in grasping the present situation due to frequent procedural changes, unclear expectations, and concerns about the availability of resources. These uncertainties forced them to operate under unstable conditions, where planning ahead often seemed futile. As Raz, a social worker and a manager at the Ministry of Health, expressed it: *How can we ensure social rights*,* welfare*,* and healthcare*,* when everything feels so uncertain? There are no positions and no staff in mental health or public health. Talking about adding resources sounds like science fiction.*

The looming threat that essential systems could fail or be compromised intensified their feelings of vulnerability and eroded their trust in the very tools meant to support their work. This created a state of continuous alertness, as they felt they had to be constantly prepared for sudden disruptions. As Avi, a doctor, explained: *It’s pressure on top of pressure*,* it’s pressure squared. Even if nothing happens*,* you’re in constant stress because you know that maybe*,* at any given moment*,* there could be a surge of patients.*

This underlying uncertainty was further exacerbated in times of severe crisis, heightening existing challenges and amplifying the emotional and operational strain on professionals. Participants described how the constant need to respond to unpredictable demands created decision fatigue, as they were required to make repeated high-stakes decisions without stable guidelines. Over time, this unpredictability contributed to an erosion of control, weakening their confidence in assessing situations and determining the best course of action. Reflecting on these extreme situations, Rani, a nurse, explained: *During both COVID and the war*,* you just didn’t know when it would end. And there weren’t enough staff… you don’t know when it will be over.*

Rather than blurring the line between routine and emergency response (as seen in Volatility), uncertainty manifested as a fundamental erosion of clarity, predictability, and control. Professionals described an inability to rely on stable procedures, consistent staffing, or clear guidelines. The absence of dependable structures meant that even routine decisions were made in an atmosphere of ambiguity, where expectations shifted and priorities remained unclear. As Dan, a doctor, reflected: *Everything becomes more extreme. There are all these narratives like ‘quiet*,* they’re shooting*,*’ meaning that during wartime*,* any conflict or disagreement that isn’t urgent should be silenced. I don’t know… it just exacerbates everything.*

Dan’s reflection demonstrates how uncertainty permeated not only clinical procedures but also the social and professional norms that guide everyday behavior. His description of a workplace where even routine disagreements must be suppressed reveals how unclear expectations and shifting priorities created confusion about what was considered acceptable conduct. This lack of clarity illustrates how uncertainty extended beyond operational tasks and into interpersonal dynamics, producing an erosion of control and contributing to the emotional strain associated with decision fatigue. As ambiguity spread across both clinical and relational domains, professionals found it increasingly difficult to anchor themselves in stable norms, further weakening their sense of confidence and stability in daily practice.

#### 4. Ambiguity - role confusion and ethical uncertainty

Many participants described how the volatility, complexity, and rapid changes within their work environment created a persistent sense of ambiguity. In this context, ambiguity refers to the lack of clarity surrounding responsibilities, procedures, and ethical standards, particularly as evolving technologies replace or alter traditional practices. In this theme, technology is discussed not as a source of rapid change, but as a factor that blurs professional boundaries and creates uncertainty regarding roles, expectations, and decision-making authority. Participants highlighted how the growing reliance on digital tools, remote care, and frequently updated protocols often blurred the boundaries of their professional roles. Rather than supporting decision-making, technological systems sometimes added confusion and undermined confidence in clinical judgment, as Dov, a doctor, explained: *the workload is increasing*,* but staffing levels stay the same. The system now relies heavily on technology*,* and let’s put it nicely*,* sometimes*,* it feels like it’s more of a trap than a help.*

A central dimension of this ambiguity was the lack of adequate guidance, supervision, and structured training accompanying technological change. Professionals reported being expected to work with new systems without clear instructions or ongoing support, leaving them to navigate ethical and clinical decisions without a stable framework. As Rama, a nurse, reflected, the absence of continued guidance left her feeling unprepared and alone: *When they first introduced the new machines*,* I didn’t know how to operate them at all… but after we finished the initial training*,* I realized I was on my own and had to solve the problems myself. You have to find a solution.*

Such experiences illustrate how technology, though intended to enhance care, can introduce ambiguity when implemented without adequate support or clear guidelines. These unclear boundaries intensified ethical uncertainty, particularly when professionals were expected to make high stakes decisions without sufficient interaction with patients or clarity about institutional expectations. Mohammad, a nurse, described the weight of this ambiguity in daily work, noting that: *in our day to day work*,* it just adds a sea of information… not all of it is relevant… the amount of information you are supposed to process is enormous.*

This ambiguity was even more pronounced during times of crisis, when the pressure to act quickly under uncertain conditions further heightened professionals’ stress and deepened their sense of vulnerability. The emotional burden of working without clear direction, especially in ethically complex scenarios, contributed significantly to their experience of moral distress.

#### 5. Conflicts between professional values and system demands

Many participants described experiences reflecting moral injury within the healthcare system.

This section explores the experience of moral injury as expressed by healthcare professionals. While elements related to moral distress appear across the previous themes, this theme focuses specifically on situations in which participants described a direct conflict between professional values and systemic constraints, reflecting experiences consistent with moral injury. The analysis reveals two interconnected categories: (1) *Tensions between professional identity and systemic structures*, and (2) *Emotional and ethical consequences of sustained strain*. Together, these themes highlight how conflicting expectations in VUCA healthcare environments generate moral distress and compromise professionals’ values and well-being.

##### 5.1 Tensions between professional identity and systemic realities

###### 5.1.1 A gap between professional values and systemic realities

Many healthcare professionals frequently described the tension between their professional commitment to high-quality, patient-centered care and the operational demands imposed by the healthcare system. Time pressures, administrative burdens, and digital documentation requirements often left them feeling as though they were compromising their professional standards. This conflict resulted in emotional distress and a sense of professional inadequacy, as they were forced to prioritize system efficiency over patient needs. As Gad, a doctor, explained: *All this overload leaves you constantly walking around with a sense of dissatisfaction*,* and a feeling that you are not doing the best you can. Many patients slip through the cracks.*

Participants described instances where they were required to enforce protocols they personally disagreed with, creating feelings of detachment from their professional values and diminishing their sense of integrity, as Dina, a nurse, expressed it: *My conflict is that I tell them to do something I don’t believe in myself. And I know I sound detached when I say it.*

The growing reliance on telemedicine for patient consultations further intensified this struggle. Some professionals expressed discomfort with making clinical decisions without physical examinations, feeling that this compromised their diagnostic accuracy and the patient-provider relationship, as one of the doctors, Abed, stated: *There are many things I wouldn’t feel comfortable deciding on without examining the patient physically… Where is the line? It depends on the provider. It’s an art.*

Economic considerations were another recurring source of moral distress. Participants recounted situations where financial pressures dictated treatment decisions, sometimes conflicting with their professional judgment about what was best for the patient: *It’s an ongoing battle between the hospitals and the HMOs. I get emails asking*,* ‘Patient X is still hospitalized—why? We’ll deduct hospitalization days from your budget.*

Many participants expressed a profound sense of powerlessness, describing how systemic constraints placed them in an ongoing struggle between adhering to institutional policies and advocating for their patients’ best interests. This tension often contributed to feelings of moral injury. Participants described the distress of being forced to prioritize administrative efficiency and budgetary concerns over their professional commitment to patient-centered care. As Nir, a nurse, explained: *On one hand*,* I need to follow protocols and guidelines*,* and on the other hand*,* the system expects all the nursing work… it becomes very technical.*

The emotional burden was particularly evident when professionals realized that systemic limitations prevented vulnerable populations from receiving adequate attention and care. They described the painful experience of witnessing marginalized groups being pushed aside, knowing that these patients were unlikely to receive support anytime soon. This inner conflict left professionals feeling torn between their understanding of the system’s urgent resource constraints and their personal drive to care for those in need. Rachel, a nurse, reflected: *Some populations*,* like individuals with mental health conditions*,* are always deprioritized. Now we’re told*,* “This isn’t the time.” But when will it ever be? Things aren’t likely to improve.*

Taken together, these accounts demonstrate how VUCA-driven systemic pressures not only strained daily operations but disrupted clinicians’ professional identity, limiting their ability to act according to the values, practices, and ethical standards that define their sense of being a healthcare professional.

###### 5.1.2 Leadership and role conflicts

Some managers described being caught between loyalty to their teams and institutional compliance. They spoke about the strain of deciding how much pressure to place on their teams to meet institutional demands, while simultaneously trying to accommodate their individual needs during difficult periods. Shay, a doctor and head of department, explained: *You try to meet both needs… the system’s needs and the employees’ needs. But the situation shifted acutely-so I was asking myself: How hard do I push them to come to work? How flexible should I be?*

Others struggled with being perceived as out of touch, like Naama, a social worker in a managerial role at a hospital: *I tell the teams*,* ‘You must do this because the regulator said so*,*’ and we check that it’s done. But it’s not logical. I force them to comply*,* and they see me as detached from reality.*

This balancing act extended beyond professional duties to personal leadership dilemmas. Ultimately, this ongoing struggle between personal values, professional expectations, and systemic constraints intensified feelings of moral injury, as professionals felt trapped in a cycle of responsibility without adequate support.

##### 5.2 Emotional toll and ethical distress

While the previous sub-theme described the structural and professional tensions that created moral conflict, this section focuses on their emotional consequences. Interviewees emphasized that the cumulative strain of working under systemic constraints and making ethically difficult choices over time resulted in significant psychological distress. Many spoke of persistent feelings of guilt, self-doubt, and emotional exhaustion, particularly in moments when they felt unable to provide the standard of care they believed their patients deserved. These emotional responses reflected not only isolated incidents but an ongoing erosion of their sense of professional efficacy and moral integrity. As Adam, a nurse, reflected: *Burnout is a very big word*,* and it doesn’t come from one incident or one shift. It’s the accumulation of fatigue*,* the accumulation of discomfort*,* the accumulation of situations that build on one another.*

###### 5.2.1 Internalizing systemic failures

The weight of systemic dysfunction is often translated into personal guilt. Professionals described moments of ethical paralysis and helplessness. Some described moments in which systemic pressures forced them to prioritize efficiency over empathy, leaving them with a lingering sense of helplessness and guilt. They recounted instances where they had to make life-or-death decisions under pressure or remain silent on issues that conflicted with their personal and professional values. These experiences left lasting emotional scars as Lily, a nurse, reflected: *There was chaos; many times*,* I didn’t know if I was doing the right thing. Once*,* I had to choose between two critically ill patients and give treatment to the one with the better survival chance. That was really tough.*

Beyond patient care, some professionals expressed moral distress arising from their inability to address broader injustices within the system. They felt torn between their values and their institutional roles, particularly when working within a political and organizational climate that discouraged open discussion of sensitive issues. As Rami, a nurse, explained:

*My conscience tells me to speak out for coexistence and against racism*,* but I can’t-the system demands silence. I felt torn apart like I was being ripped in two. I felt… torn apart inside. I don’t even know how to explain it-it felt like being ripped in two… Where am I in all of this?*

###### 5.2.2 The burden of personal responsibility

Despite understanding system limitations, participants often felt personally accountable for patient outcomes. The expectation to suppress emotion in favor of professionalism added to this burden, leading to guilt, frustration, and moral discomfort. Professionals described an internal struggle between empathy and the clinical detachment required of them: *You are a professional*,* but on a human level… you are affected. But I know I would feel worse if I acted emotionally and not according to my professional values.*

Despite knowing that health inequality and staffing shortages were systemic problems, participants often internalized these failures as personal shortcomings. They felt responsible for patients who were disadvantaged by the system, even when structural barriers made proper care impossible. As Raz, a social worker with managerial responsibilities at a hospital, explained: *The issue of health inequality deeply concerns me… I came into this role for that reason. But when there aren’t enough positions*,* I feel like I’m failing…*.

###### 5.2.3 Blurring of boundaries between work responsibilities and personal well-being 

Healthcare professionals described a persistent struggle to maintain boundaries between their professional responsibilities and personal well-being. This tension was evident in their working hours, physical safety concerns, and the psychological burden of balancing work obligations with personal and family commitments.

The expectation of constant availability to patients, colleagues, and the organization eroded work-life boundaries, particularly with the expansion of remote work, digital communication, and the demand to remain responsive beyond formal working hours. Professionals reported sacrificing their breaks and arriving early to fulfill their responsibilities, often without additional compensation. Liat a doctor shared: *There are no work hours-I go to work*,* come home*,* and continue working on the computer. On one hand*,* there’s accessibility; on the other*,* it’s hard to find balance.* Another nurse added: *I find myself giving up my break just to get everything done… We have nurses who arrive half an hour early-before 7 a.m.*,* meaning they get here at 6:30-just to get things ready*,* and they’re not paid for that extra time*.

During health crises, such as the COVID-19 pandemic, these blurred boundaries intensified. Professionals grappled with fears of contracting the virus and infecting their families, creating a profound emotional burden as they tried to balance their duty to patients with the need to protect loved ones. David, a nurse, explained: *At the start of COVID*,* before the vaccines*,* we were afraid we’d get infected and die.*

Similarly, in crisis situations like war, professionals face acute dilemmas regarding their physical presence. They were forced to choose between prioritizing patients or ensuring their families’ safety. This internal conflict between professional duty and personal security caused significant moral strain, as one of the nurses, Rim, explained: *The first dilemma was: home or work? I’m in a bomb shelter now. Do I get in the car and go to work or stay with my kids? Who needs me more?*

These quotations highlight the ongoing struggle professionals face when determining where their primary obligations lie toward their patients, their families, or their own well-being. The absence of clear boundaries compounded feelings of guilt, anxiety, and exhaustion, ultimately contributing to moral injury and emotional distress.

#### 6. Normalization of crisis as a coping strategy

Numerous participants described ongoing exposure to high-pressure situations, compounded by workforce shortages and overwhelming workloads, has resulted in a state of persistent strain. Participants described this condition as a “chronic crisis,” where heightened stress became normalized as part of their professional reality. This reflects a coping pattern best described as a normalization of crisis within VUCA conditions, in which continuous exposure to emergencies leads professionals to adapt by treating extreme situations as routine. Raviv, a doctor, noted that emergency scenarios have become so routine that they no longer evoke a heightened response: *Emergency situations*,* especially in the ER*,* are now just routine. It almost doesn’t feel like an emergency anymore.*

Despite the unpredictability of their environment, professionals paradoxically described finding stability in the constancy of this pressure. The knowledge that the work would always be demanding created a “safe bubble” within the broader uncertainty. Nilli, a nurse, explained: *We need more staff and more resources-the system is intense and demanding. There’s always work*,* always patients seeking help. But this system is well-oiled; it always functions-during war*,* illness*,* COVID-it’s constant. You’re not worried it’ll stop. That’s certain.*

This perceived stability, however, came at a cost. The normalization of extreme stress led to the internalization of crisis as the default state, embedding high tension into their daily routines. Over time, this adaptive pattern produced unintended emotional consequences. Professionals described bringing this accumulated stress into their personal lives, affecting their emotional well-being and their relationships with patients and families. As one of the nurses, Afnan explained: *and then it carries over… people bring it to work*,* to the patients. In the end*,* how do you hold yourself together? How do you hold the patients? How do you hold your kids?*

Ultimately, this normalization of crisis conditions enabled professionals to maintain functionality in an unpredictable environment; however, it also desensitized them to the long-term emotional strain, further entrenching the conditions that contribute to moral injury.

## Discussion

The findings of this study underscore that moral injury among healthcare professionals is not merely an individual psychological response, but a consequence of systemic stressors shaped by broader structural conditions. Using the VUCA framework as an analytical lens, this study highlights how macro-level characteristics, volatility, uncertainty, complexity, and ambiguity, directly influence clinical practice and ethical integrity, particularly in hospitals that are first to absorb the consequences of systemic instability.

Volatility and complexity were experienced as an ongoing “state of emergency,” with professionals describing constant changes in policies, patient surges, and technological adjustments, leaving them in a state of continuous alertness and strain. This aligns with previous research highlighting the normalization of crisis conditions in healthcare under systemic pressure [3, 45], and the current study extends this insight by showing how such chronic instability not only shapes operational routines but also becomes a central pathway through which moral injury develops.

Uncertainty permeated their daily operations, as professionals struggled to adapt to unpredictable shifts and feared technological failures, fostering decision fatigue and eroding their sense of control [69, 71]. Ambiguity further compounded this stress, with blurred boundaries in professional roles and ethical responsibilities, particularly with remote care and digital systems, leaving professionals uncertain about their clinical judgment and accountability [25]. Complexity, particularly in the digitalization of healthcare and multi-level bureaucracies, complicates task execution and dilutes provider-patient interaction. Although innovation can increase efficiency, the ethical cost arises when systems prioritize data and protocols over human-centered care [15, 17] (Naamati-Schneider, 2023; Pogorzelska & Chlabicz, 2022). These operational frictions compromise clinicians’ ethical ideals and deepen feelings of disconnection from their core values.

These VUCA conditions disrupted the alignment between professional values and system demands. Professionals described the emotional toll of prioritizing administrative efficiency over patient-centered care due to financial and resource constraints, leading to feelings of guilt, frustration, and detachment from their professional identity [82].

A central theme was the burden of personal responsibility, which further intensified the emotional toll. Even when aware of systemic limitations, professionals internalized adverse outcomes as personal failures. Many described being caught between institutional constraints and their ethical commitment to patients—consistent with moral injury literature that highlights guilt, shame, and helplessness when professionals are unable to act in accordance with their values [56, 59, 69]. The contribution of this study lies in demonstrating that within VUCA healthcare environments, personal responsibility becomes multidimensional. Professionals described feeling simultaneously accountable to patients, colleagues, and their own families, resulting in a layered moral tension that extends beyond clinical care. This expanded sense of responsibility intensified ethical strain, particularly when individuals were required to prioritize organizational directives over the needs of patients or staff. Managers faced an additional burden, navigating the conflict between maintaining institutional compliance and supporting their teams, thus experiencing moral pressure from both directions. This nuanced understanding highlights how moral injury is shaped not only by clinical decisions but also by the complex interplay of professional, relational, and organizational obligations in volatile and uncertain healthcare settings.

This emotional strain was further heightened in crisis settings, such as war or the COVID-19 pandemic, where professionals not only faced increased workloads but also had to navigate personal safety concerns and the welfare of their families. These overlapping pressures reflect the challenges described in research on the impact of emergencies and high-stress environments on moral injury among healthcare workers [67, 70]. The present study extends this literature by showing how these crisis-related pressures interact with the everyday VUCA conditions that already shape clinical work. Rather than treating crisis events as isolated episodes, the findings demonstrate that professionals experience them as intensifications of an ongoing structural instability. This continuity between routine conditions and emergency situations reveals a cumulative form of moral strain that is not adequately captured in existing research. Moreover, the study highlights how crises amplify existing ambiguities in roles, expectations, and ethical responsibilities, exposing professionals to multiple, simultaneous layers of vulnerability that deepen moral injury in ways not previously described.

The erosion of boundaries between professional and personal life further compounded this burden. Professionals described how the demand for constant availability, remote work, and emergency conditions dissolved the separation between work and home. The expectation to prioritize work, even at the expense of their own well-being, left many questioning how to balance their duty toward patients with their responsibility to themselves and their families. This finding is consistent with studies highlighting the growing challenge of maintaining work-life balance in digital and crisis-affected healthcare environments [37]. This study advances existing knowledge by showing that the blurring of boundaries is not merely a challenge of time management or digital overload, but a mechanism through which moral injury deepens. The findings demonstrate that when work intrudes into personal life under VUCA conditions, professionals do not simply experience fatigue; they experience an erosion of their ability to uphold their own ethical standards in both domains. The study further adds that the collapse of boundaries intensifies identity conflicts, forcing professionals to continuously renegotiate where their primary obligations lie.

Finally, the normalization of crisis conditions emerged as both a coping mechanism and a source of harm. Participants described a paradoxical stability within the chaos-the certainty that work would always be intense and demanding created a “safe bubble” of predictability. However, this normalization of stress reinforced a culture in which extreme pressure was accepted as routine, making it harder for professionals to acknowledge or seek help for the emotional toll it inflicted. This entrenchment of crisis as the default condition further perpetuated moral injury, as professionals continued to operate under unsustainable conditions without adequate systemic support. This finding aligns with research on “chronic crisis” in healthcare settings, which highlights the normalization of stress and the long-term implications for professional well-being [45].

Overall, the study suggests that moral injury in healthcare is not simply an individual experience but is embedded in the structural and cultural realities of the healthcare system. The VUCA framework helps illuminate how volatility, uncertainty, complexity, and ambiguity shape not only the conditions under which professionals work but also their internal responses: distorting control, weakening ethical confidence, and forcing difficult compromises. This supports previous arguments that moral injury stems from systemic pressures more than personal weakness, and that individual-focused interventions may be insufficient [65–66]. This is particularly important in the healthcare field because when professionals’ ethical confidence and decision-making capacity are compromised, the consequences extend beyond the individual worker and directly affect patient safety, quality of care, and organizational trust. Understanding moral injury as a systemic phenomenon therefore allows healthcare institutions to identify structural risks that jeopardize both workforce well-being and patient outcomes.

Recognizing this connection between systemic VUCA pressures and moral injury is crucial for developing interventions that address both the external conditions and the internal struggles of healthcare professionals. Efforts to strengthen resilience must go beyond individual coping strategies and focus on creating organizational environments that reduce volatility, clarify procedures, and support ethical decision-making. Without addressing these underlying systemic pressures, healthcare professionals will continue to bear the emotional and ethical cost of functioning in an unpredictable environment. As noted in prior work, institutional support, ethical leadership, and organizational changes that prioritize professional values alongside operational efficiency are essential for safeguarding both healthcare professionals’ well-being and the quality of patient care [46, 82].

### Implications for policy and practice

The findings emphasize the need to shift from interventions centered solely on individual resilience to broader systemic reforms that address the structural drivers of moral injury. Healthcare professionals operate in a VUCA environment that not only heightens ethical tensions but frequently forces professionals to compromise their core values [3, 6]. Placing the burden on individuals to endure these pressures risks exacerbating their moral distress and deepening their sense of powerlessness.

Promoting workforce well-being requires a comprehensive approach that aligns system demands with professional values. This includes reducing administrative burdens, allowing realistic adaptation periods for new technologies, and safeguarding time for rest and recovery. Integrating Industry 4.0 technologies must be accompanied by adaptive leadership and regulatory preparedness to mitigate disruption [83]. Healthcare organizations should establish safe spaces where professionals can raise ethical concerns without fear of repercussions and train leaders to identify and address moral injury as distinct from burnout. Strengthening this alignment between organizational systems and professional ethics is crucial to protecting both the well-being of healthcare professionals and the quality of patient care.

### Study limitations and directions for future research

This study offers valuable insights into moral injury among healthcare professionals in VUCA environments, yet several limitations should be noted. As a qualitative study focused on the Israeli context, the findings may not be fully generalizable to other healthcare systems. Broader cross-cultural research is needed to assess whether similar patterns exist in other regions or other crisis-affected systems.

The reliance on self-reported narratives may introduce recall or social desirability bias. Future studies could benefit from incorporating additional data sources, such as observations or institutional documents. While participants came from diverse roles, this study did not systematically compare responses by age, seniority, or profession. Future research should explore how these factors, and intersectional dimensions like gender or ethnicity, shape the experience of moral injury.

Lastly, further work is needed to refine the VUCA framework in healthcare and examine how its elements evolve across different types of crises. Given that volatility, uncertainty, complexity, and ambiguity may manifest differently across different healthcare settings (e.g., primary care, long-term care, emergency medicine, or community-based services), future research should investigate how VUCA conditions operate in these diverse environments. Such comparative work would clarify whether the mechanisms identified in this study reflect broader patterns within healthcare or are specific to particular organizational or systemic contexts. Longitudinal and comparative studies would help clarify the long-term impact of moral injury and support the development of effective system-level interventions.

## Conclusion

This study highlights the profound impact of VUCA-driven pressures on healthcare professionals, demonstrating how systemic volatility, uncertainty, complexity, and ambiguity contribute to moral injury. The tension between professional values and operational constraints generates emotional and ethical distress, eroding professionals’ sense of control and integrity. These findings underscore the need for healthcare organizations to move beyond individual-focused solutions and address the systemic conditions that jeopardize both professional well-being and patient care. Creating environments that align institutional processes with healthcare professionals’ core values is essential for safeguarding both the workforce and the quality of care in an increasingly complex healthcare landscape.

## Supplementary Information

Below is the link to the electronic supplementary material.

Supplementary Material 1

## Data Availability

Data are available upon request.
