# Supplementary material for: Moral injury in a VUCA healthcare environment: ethical complexities and professional challenges for medical teams
Source: BMC Health Serv Res. 2026 May 8;26:899. doi: 10.1186/s12913-026-14664-2 (PMC13325781; doi:10.1186/s12913-026-14664-2)
Supplement: Supplementary file 1 — Supplementary Material 1 [file 12913_2026_14664_MOESM1_ESM.docx]

**Semi-Structured Interview Guide**

**Interview conducted as part of a study on patient experience and moral injury among healthcare professionals**

**Participant background information**
Affiliated organization (medical center / healthcare organization): _____________________
Participant name or pseudonym: _____________________
Professional role: _____________________
Department / unit: _____________________

**1. Opening question**
Please share the associations or thoughts that come to mind when you think about the healthcare system.

____________________________________________________________________________________________________________________________________________________________________________________________________________________________________________________________________________________

**2. Personal and systemic conflicts**
Please describe the personal or systemic conflicts that healthcare teams encounter in their work within healthcare organizations.

____________________________________________________________________________________________________________________________________________________________________________________________________________________________________________________________________________________

*If not addressed spontaneously, please probe the following areas:*

- Ethical problems or value conflicts
- Moral dissonance or double standards (situations where one thing is said, but another is done or expected in practice)
- Patient prioritization or selection in care or treatment
- Ethical dilemmas encountered in daily work

**3. Personal and moral experiences**
Please describe your personal and value-based experiences during your work in a hospital or clinic. You may refer to personal experiences, difficulties, moments of uncertainty, or situations that caused discomfort.

____________________________________________________________________________________________________________________________________________________________________________________________________________________________________________________________________________________

*If not addressed spontaneously, please probe the following areas:*

- A meaningful or memorable experience related to patient care or service
- An example of a situation in which you would have acted differently if the system had allowed it
- Ways in which the system or colleagues led you to act in ways that conflicted with your personal or professional values
- Situations in which you felt guilt
- Situations in which you felt shame
- Changes you believe would improve your work experience or well-being

**4. Commitment to the system**
What keeps you working within the healthcare system?

____________________________________________________________________________________________________________________________________________________________________________________________________________________________________________________________________________________

**5. Closing question**
Is there anything else you would like to add, or any message you would like to convey to healthcare policymakers that we have not discussed?

____________________________________________________________________________________________________________________________________________________________________________________________________________________________________________________________________________________
